# ABCD transfer matrix model of Gaussian beam propagation in Fabry-Perot etalons

**DOI:** 10.1364/OE.477563

**Published:** 2022-12-06

**Authors:** David Martin-Sanchez, Jing Li, Dylan M. Marques, Edward Z. Zhang, Peter R. T. Munro, Paul C. Beard, James A. Guggenheim

**Affiliations:** 1 Department of Medical Physics and Biomedical Engineering, University College London, UK; 2 Institute of Cardiovascular Sciences, College of Medical and Dental Sciences, University of Birmingham, UK; 3 Wellcome / EPSRC Centre for Interventional and Surgical Sciences, University College London, UK; 4 School of Computer Science, College of Engineering and Physical Sciences, University of Birmingham, UK

## Abstract

A numerical model of Gaussian beam propagation in planar Fabry-Perot (FP) etalons is presented. The model is based on the ABCD transfer matrix method. This method is easy to use and interpret, and readily connects models of lenses, mirrors, fibres and other optics to aid simulating complex multi-component etalon systems. To validate the etalon model, its predictions were verified using a previously validated model based on Fourier optics. To demonstrate its utility, three different etalon systems were simulated. The results suggest the model is valid and versatile and could aid in designing and understanding a range of systems containing planar FP etalons. The method could be extended to model higher order beams, other FP type devices such as plano-concave resonators, and more complex etalon systems such as those involving tilted components.

## 1. Introduction

Fabry-Perot (FP) etalons are optical cavities comprising a thin spacer sandwiched between two planar mirrors. These devices are used as filters and sensors for a range of applications including spectroscopy [1], multiplexing [2], ultrasound detection [3], and temperature measurement [4].

A key characteristic of FP etalon systems is the wavelength resolved interferometer transfer function (ITF). This ITF quantifies the light intensity transmitted or reflected by the etalon as a function of the optical wavelength. The ITF exhibits a series of sharp, periodic interference fringes, the characteristics of which usually have a significant impact upon the system performance. In filters for example, the fringes define the passbands. In sensors, their gradients and visibilities determine the sensitivity [5]. As such, predicting the ITF can aid the design and evaluation of etalon based systems.

Predicting the ITF requires calculating the fields reflected and transmitted by the etalon, accounting for the fact that light makes multiple round trips in the cavity and produces multiple partial beams which interfere. In principle, the partial beams can be predicted, then summed to provide the total field. If the etalon is illuminated by a plane wave, the partial beams can be expressed as a geometric series and summed to yield the well-known Airy function (AF) [6]. The closed form nature of the AF means its computational implementation can be highly efficient. However, the AF is inaccurate if the etalon is illuminated by a focussed beam that is poorly approximated by a plane wave [7]. In these circumstances, predicting ITFs requires a more sophisticated approach. One such approach exploits the angular spectrum method rooted in Fourier optics to broaden the applicability of the AF to focussed beams [8]. In the resulting so-called “Angular Airy Function” (AAF) approach, the beam is decomposed into an angular spectrum of plane waves. The field produced by each plane wave propagating through the etalon is calculated independently using the AF and then summed to provide the total field [7,9]. This angular spectrum based approach has also been applied with a multi-layer interference model in place of the AF to propagate the plane waves while accounting for the optical thicknesses of all the layers forming the etalon [10].

Other FP etalon models are based on “unfolding the FP cavity”. This entails iteratively calculating the beam profile after successive round trips by analytically propagating the beam the required distance through the spacer, then summing the resulting partial beams [11,12]. This approach is more computationally expensive than AF-based methods.

A common disadvantage of both the angular spectrum and unfolded cavity approaches is they are not always amenable to being integrated with models of other optical components in the system that influence the ITF, for example, the lenses used to shape the optical beam that illuminates the etalon.

To provide an alternative model that overcomes this challenge, a model of etalon systems based on the ABCD transfer matrix method [13] was developed. With this method, the components in an optical system are each represented by so-called “ABCD” matrices. The entire system response is then given by the product of these matrices. The ABCD method is widely used and has already been applied to study kilometre scale, concave FP interferometers used to detect gravitational waves [14]. Here, it is applied to model systems containing planar FP etalons. To enable this, an ABCD etalon matrix is defined and then multiplied by the ABCD matrices representing the optical components that deliver light to and from the etalon in order to model the whole system. One advantage of this approach is that, as well as describing the etalon, the ABCD formulation can easily describe an arbitrary number of optical elements delivering the light to and from the etalon. The approach thus lends itself to modelling entire etalon based systems within a common methodological framework in a way that is challenging with other approaches. To validate the resulting ABCD etalon model, simulated fields and ITFs were compared with those calculated using a previously validated AAF model [7]. To demonstrate the new model, simulations of three different etalon system were performed. The results suggest the model is valid and versatile and could help with designing and evaluating a range of etalon-based systems.

The following sections describe the concept and implementation of the model, its validation, demonstrations, and then discussions and conclusions.

## 2. Model

This section describes the ABCD model of Gaussian beam propagation through FP etalons. First, the ABCD framework, existing ABCD matrices, and etalon matrix are described. Then, the methods for calculating the optical fields and ITFs in an etalon system are explained.

### 2.1 ABCD method

The ABCD transfer matrix method simulates the propagation of paraxial beams such as plane waves and Gaussian beams through an optical system. The system is described by a series of so-called “ABCD” matrices, each of which represents the propagation of light through an optical component, or set of components, along an optical axis, *z*, defined by the trajectory of the beam. The matrices propagate the beam as

(1) q(z1)→[ABCD]→q(z)=Aq(z1)+BCq(z1)+D

where the beam is described by a term *q(z)*, known as the complex beam parameter [15]. This parameter is a complex number describing the profile of the beam at a plane normal to the optical axis. For a Gaussian beam,

(2) $$\frac{1}{q(z)}=\frac{1}{R(z)}-i\frac{2}{k\omega^{2}(z)},$$

where *R(z)* is the radius of curvature, *ω(z)* is the *1/e^2^* beam width, *k = 2πn/λ* is the wave number, *n* is the refractive index of the medium, and *λ* is the wavelength of the beam. At the beam waist, the complex beam parameter is purely imaginary and 1/*q_0_=−2i/kω_0_^2^*, where *ω_0_* is the *1/e^2^* focal spot size and the curvature term *R_0_* is infinite indicating a planar wavefront.

Equation (1) is known as the “ABCD law”, and it is used to analytically determine the profile of an incident Gaussian beam after it has propagated through an optical system, as a solution of the paraxial wave approximation [16]. Considering the lowest-order mode, the complex amplitude of the reflected or transmitted field, *U_out_*, across the radial axis, *r*, at a position *z* along the optical axis, after propagating through the optical system described by *M = [A B;C D]*, is defined as

(3) $$U_{out}(r,z;M)=\frac{1}{A+B/q(z_{1})}\exp(-ik\frac{r^{2}}{2q(z)}),$$

where the planes *z_1_* and *z* are located just before and after the optical system, respectively. Substituting Eq. (2) into Eq. (3) leads to the more familiar expression of the complex amplitude of a fundamental Gaussian beam [17]

(4) $$U_{out}(r,z)=\frac{\omega_{0}}{\omega (z)}\exp(-\frac{r^{2}}{\omega^{2}(z)})\exp(-ikz-\frac{ikr^{2}}{2R(z)}-i\zeta (z)),$$

where *ς=-tan^−1^(z/z_0_)* is the Gouy phase, *z_0_ = kω_0_^2^/2* is the Rayleigh range and *ω_0_* is the beam waist.

Equation (4) allows calculating the beam characteristics after propagation through any optical system or component represented by an ABCD matrix. As the ABCD method is well-established, existing ABCD matrices have already been defined for a wide range of optical elements including homogeneous space, thin lenses, graded index (GRIN) lenses, and mirrors. A list of examples are provided in Table 1 [18].

**Table 1.** ABCD matrices of some optical elements [18].

Description	Representation	Matrix
Propagation through homogeneous medium	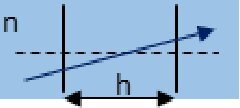	$M_{prop}=\left[\begin{array}{cc} 1 & h/n\\ 0 & 1 \end{array}\right]$
Refraction at a planar boundary	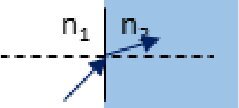	$M_{refr}=\left[\begin{array}{cc} 1 & 0\\ 0 & n_{1}/n_{2} \end{array}\right]$
Refraction at a spherical boundary	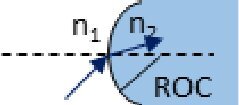	$M_{sph\_refr}=\left[\begin{array}{cc} 1 & 0\\ -(n_{1}-n_{2})/n_{2}ROC & n_{1}/n_{2} \end{array}\right]$
Transmission through a thin lens	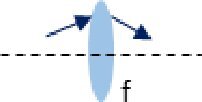	$M_{lens}=\left[\begin{array}{cc} 1 & 0\\ -1/f & 1 \end{array}\right]$
Reflection from a planar mirror	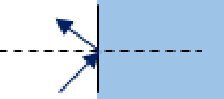	$M_{refl}=\left[\begin{array}{cc} 1 & 0\\ 0 & 1 \end{array}\right]$
Reflection from a spherical mirror	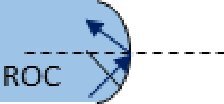	$M_{sph\_refl}=\left[\begin{array}{cc} 1 & 0\\ 2/ROC & 1 \end{array}\right]$
Propagation through radial gradient index (GRIN) medium	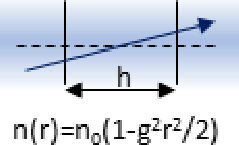	$M_{GRN}=\left[\begin{array}{cc} \cos(gh) & \sin(gh)/g\\ -g\;\sin(gh) & \cos(gh) \end{array}\right]$

As illustrated by the diagrams in the middle column of Table 1, ABCD matrices propagate a beam in one direction through an optical component. Propagating the beam in the opposite direction requires a matrix, *M^←^*, found by swapping the *A* and *D* matrix elements of *M* [19].

As the ABCD matrices are linear operators, the propagation of light through an optical system comprising any number of components can be represented by a single ABCD matrix found by multiplying the individual component matrices together in sequence. This is illustrated in Eq. (5), in which the right-most matrix corresponds to the first optical component encountered by the beam.

(5) $$M=M_{N}\cdots M_{2}M_{1}$$

Once the optical system is represented by an ABCD matrix, Eq. (3) can be used to simulate beam propagation through the system. As the ABCD formalism does not account for changes in amplitude however, an additional operation is required to consider certain components such as partially reflective mirrors, as described in section 2.2.3.

### 2.2 FP etalon model

Propagating a Gaussian beam through an optical system containing an FP etalon using the ABCD method requires: (1) representing the propagation of a beam through the etalon using an ABCD matrix; (2) multiplying the etalon matrix by the matrices representing the optical components delivering light to and from the etalon to calculate a system matrix (3); propagating partial beams through the system using the system matrix; (4) summing the resulting fields. By repeating this procedure for different wavelengths, the ITF can be computed.

#### 2.2.1 Representing the etalon

**Fig. 1.**
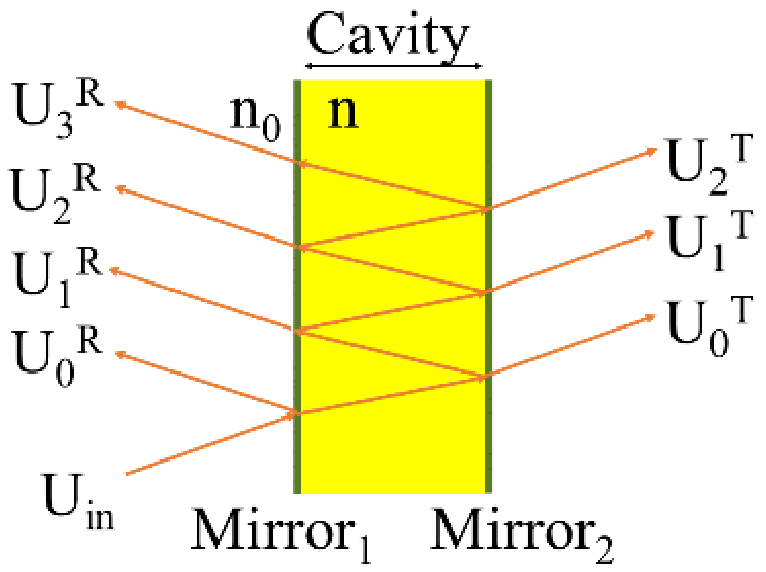
Multiple-beam interference in a Fabry-Perot etalon. Note, normally-incident beams are assumed in this work; the diagonal rays are for illustrative purpose only.

An FP etalon consists of two planar, parallel mirrors of reflectivity *R_1_* and *R_2_*, separated by a spacer of thickness *h* and refractive index *n* as shown in Fig. 1. An incident beam, *U_in_*, illuminating the etalon at normal incidence generates multiple partial beams, *U_m_*, each exiting the cavity after undergoing a number of round trips, *m*, inside the cavity. In reflection mode, the output beam is the result of the interference of the partial beams exiting the first mirror (*U_1_^R^*, *U_2_^R^*, *U_3_^R^*,…) and the light reflected by the first mirror (*U_0_^R^*). In transmission mode, the output beam is the interference of the partial beams transmitted through the second mirror (*U_0_^T^*, *U_1_^T^*, *U_2_^T^*,…). An accurate model of the etalon must faithfully represent this behaviour by accurately calculating and summing the partial beams.

To represent an FP etalon using ABCD matrices, three matrices are needed to propagate the beam: (1) into the cavity, *M_in_*; (2) through one round trip, *M_cav_*; and (3) out of the cavity, *M_out_*. Propagation into the cavity simply involves a refraction by the first mirror,

(6) $$M_{in}=M_{refr}.$$

The round trip consists of a propagation through the cavity, reflection at the second mirror, reversed propagation through the cavity, and reflection at the first mirror,

(7) $$M_{cav}=M_{refl}^{\leftarrow }M_{prop}^{\leftarrow }M_{refl}M_{prop}.$$

The exit matrix *M_out_* depends on whether reflection or transmission is considered. In transmission mode, the beam propagates through the cavity and is then refracted by the second mirror. In reflection mode, the beam travels half a round trip and is then refracted by the first mirror,

(8) $$M_{out}=\{\begin{array}{cc} M_{refr}M_{prop}, & transmission\\ M_{refr}^{\leftarrow }M_{prop}^{\leftarrow }M_{refl}M_{prop}, & reflection \end{array}$$

Combining these operations, the ABCD matrix of the FP etalon becomes

(9) $$M_{FP,m}=M_{out}(M_{cav}{)}^{m}M_{in}.$$

In transmission mode, Eq. (9) holds for any partial beam *m ≥ 0.* In reflection mode, Eq. (9) holds for all partial beams *m > 0*, while the first partial beam (*m = 0*) is defined as *M_FP,0_ = M_refl_*.

#### 2.2.2 Calculating the system matrix

To represent the propagation of each partial beam in a system containing an etalon and other optical components, the system ABCD matrix is given as

(10) $$M_{syst,m}=M_{\text{detection}}M_{FP,m}M_{\text{illumination}},$$

where *M_illumination_* is the matrix representing those elements between the light source and the etalon and *M_detection_* represents the components between the etalon and the detector.

#### 2.2.3 Calculating the transmitted and reflected fields

Once the system matrix is defined [Eq. (10)], the profile of each partial beam, *U_m_*, can be obtained using Eq. (4). Then, the total output field, *U_out_*, is calculated by summing the partial beams produced on all the round trips, accounting for the transmission and reflection coefficients of the etalon mirrors

(11) $$U_{out}(r,z)=\sum_{m=0}^{\infty }A_{m}U_{m}(r,z;M_{syst,m}),\;A_{m}=\{\begin{array}{cc} t_{1}t_{2}{\left(r_{1}r_{2}\right)}^{m} & m\geq 0,transmission\\ t_{1}r_{2}t_{1}{\left(r_{1}r_{2}\right)}^{m-1} & m>0,reflection\\ r_{1} & m=0,reflection \end{array}$$

where *r* and *t* are the amplitude reflection and transmission coefficients of the mirrors of the etalon, and the subindices ‘1’ and ‘2’ refer to the first and second mirrors respectively [20].

### 2.3 ITF calculation

The ITF is defined as

(12) $$ITF(\lambda )=\frac{I_{out}(\lambda )}{I_{in}(\lambda )},$$

where $I_{out}$ is the light intensity measured by a detector after the light has propagated through the etalon system. To compute $I_{out}$ , the output optical field must be weighted to account for the spatial response of the detector and integrated over its aperture. Two common detection schemes involve (1) using a uniformly sensitive detector that is larger than the beam thus rendering the detector effectively infinite in extent and sensitive only to intensity [21,22]; (2) coupling light into a detector via a single-mode fibre, rendering the detector small and phase-sensitive [23,24]. In the first case (large detector), the measured intensity is the spatial integration of the field

(13) $$I_{out}=\int_{0}^{\infty }{|U_{out}(r,z)|}^{2}2\pi rdr,$$

where *r* represents the radial axis. In the second case (single mode fibre), the field is spatially filtered by the finite size of the fibre core then integrated giving [25]

(14) $$I_{out}={\left|\int_{0}^{\infty }U_{out}(r,z)\exp(-\frac{4r^{2}}{MFD^{2}})2\pi rdr\right|}^{2},$$

where *MFD* is the mode field diameter of the fibre.

## 3. Validation

To validate the ABCD FP etalon model, it was compared to the experimentally well-validated Angular Airy function (AAF) model described in Ref. [7], in two sets of simulations. For consistency with Ref. [10], these simulations made use of Gaussian beams with focal beam waists in the range 30-250 µm, and 102 µm thick fused silica etalons of variable mirror reflectivity. As in Ref. [10], the AAF simulations were performed using the software package Jolab [26] and the data and parameters were identical to those in that paper.

The first set of simulations involved comparing the reflected fields (*|U_out_|*) produced by an FP etalon illuminated by a Gaussian beam, as predicted by the ABCD and AAF models. An optical wavelength of 1552 nm was chosen to match a resonance wavelength of the etalon. At this wavelength, the etalon had a mirror reflectivity of 97%. The simulations were done with a Gaussian beam with a focal beam waist of 50 µm, then repeated with a second Gaussian beam with a focal beam waist of 250 µm. In principle, calculating the total field requires summing over an infinite number of round trips [Eq. (11)]. In practice, it required summing over a finite number of round trips, after which adding more round trips had no impact. As such, it was necessary to dynamically identify the number of round trips after which the total field had converged. It was empirically decided to consider the model to have converged when the sum of the change in the field amplitude due to the latest round trip was <0.001%.

**Fig. 2.**
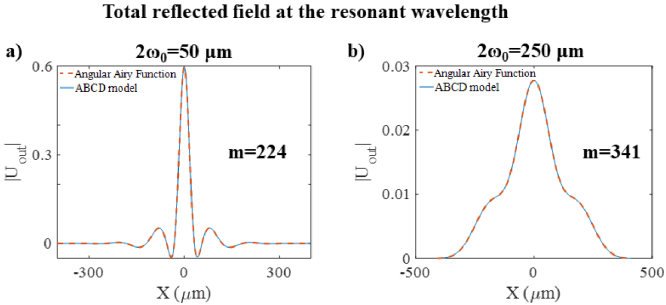
Total reflected field, *|U_out_|*, calculated using the ABCD model (blue line) as compared to the AAF (red dashed line) for an FP etalon (h = 102 µm, n = 1.444, R_1_ = R_2_ = 97%) illuminated at normal incidence by a Gaussian beam with a spot size of (a) 2ω_0_ = 50 µm (top row) and (b) 2ω_0_ = 250 µm, using 200 radial spatial sampling points, indicating the number of partial beams calculated before convergence. Simulations performed at the resonance wavelength.

After the total reflected fields were calculated using the ABCD model, line profiles through the resulting field distributions were plotted in Fig. 2, along with those calculated using the AAF for comparison. For the more tightly focussed beam [ Fig. 2(a)], the AAF predicts a tightly peaked amplitude profile with two small side lobes. For the more weakly focussed beam [Fig. 2(b)], the AAF model predicts a more broadly peaked profile with a significantly lower amplitude as expected due to the better beam confinement. For both beams, the profiles predicted by the ABCD model are in excellent agreement. Quantifying this agreement, the total root mean square difference between the profiles provided by the two models was <1% for both beams.

**Fig. 3.**
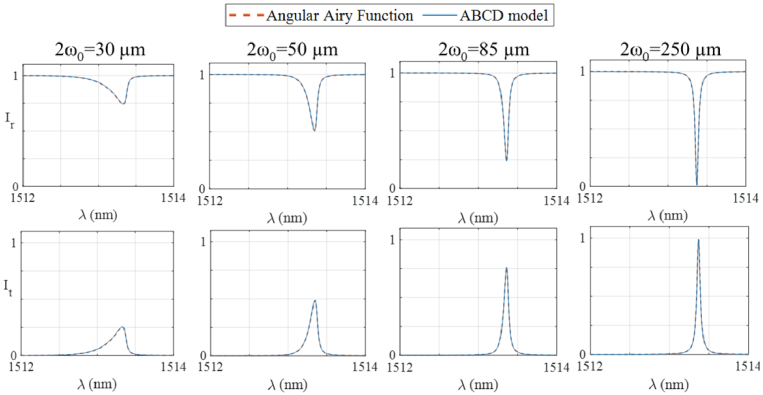
ITFs of an FP etalon (h = 102 µm, n = 1.444, R_1_ = R_2_ = 97%) calculated using the Angular Airy Function model (dashed lines) and the ABCD model (solid lines) illuminated at normal incidence by four Gaussian beams of different focal spot size (2ω_0_) as per the figure subtitles. The ITFs were simulated in reflection mode (top row) and transmission mode (bottom row). An infinitely large detector was assumed, and the infinite extent of the field was approximated by 10ω_0_. The spectral sampling interval rate was 1 pm and the radial profile was sampled using 200 radial spatial sampling points.

The second set of simulations involved comparing ITFs predicted using the ABCD and AAF models. In the first of these simulations, the same FP etalon of 97% reflectivity introduced above was illuminated by a Gaussian beam with a 30 µm focal beam waist. An ABCD model was constructed, and reflection and transmission mode ITFs were calculated using Eq. (13). The corresponding ITFs predicted by the AAF were then calculated as described in Ref. [7]. The resulting ITFs were plotted in Fig. 3 (left-most subfigures). In the plotted wavelength range, the reflection mode ITF predicted by the AAF (top subfigure) contains a single, relatively shallow and asymmetric interference fringe. As expected, the transmission mode ITF is inverted but otherwise similar. In both cases, the ITFs predicted by the ABCD model are in excellent visual agreement. The simulations were repeated for three other Gaussian beams with focal beam waists of 50, 85 and 250 µm, and the resulting ITFs were plotted in Fig. (3). As the focal beam waist increased, the ITFs predicted by the AAF had increasing depth and symmetry, reflecting the better beam confinement in the etalon. As before, the ITFs predicted by the ABCD model are in excellent visual agreement in all cases.

**Fig. 4.**
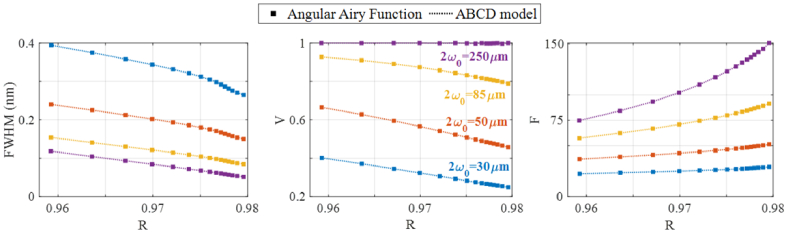
Comparison of fringe width (left), visibility (centre) and finesse (right) simulated with the Angular Airy Function (dots) and the ABCD model (lines) using several spot sizes considering an infinite aperture detector. FP etalon made of fused silica (n = 1.444 at λ=1550 nm), h = 102 µm, and variable mirror reflectivities, *R_1_* = *R_2_* between 95.8% and 98%. Visibility is defined as V=|I_max_-I_min_|/(I_max_ + I_min_) and finesse as the free spectral range divided by the FWHM.

To quantitatively compare the ITFs predicted by the models more comprehensively, the ITF simulations above were repeated for a range of different etalon mirror reflectivities. Afterwards, three metrics, namely the full width half maximum (*FWHM*), visibility (*V*) and finesse (*F*) of the resulting ITFs were compared. These metrics were calculated and plotted in Fig. 4 as a function of the etalon reflectivity. Considering first the predictions of the AAF, the FWHM decreases and the finesse increases with increasing reflectivity, in line with the increasing Q-factor of the etalon. The FWHM is also lower, and the finesse higher, for the beams of larger beam waist, due to the better confinement of the larger beams in the etalon. For most of the beams, the visibility decreases with increasing reflectivity. The exception is the 250 µm beam, for which the visibility is close to 1 for all reflectivities. Across the full range of all three metrics, the values extracted from the ITFs predicted by the ABCD model are in excellent agreement with those predicted by the AAF, with an average error below 1%. This minor residual difference was attributed to numerical error.

Overall, both the fields and the ITFs calculated using the ABCD and AAF etalon models were near identical for a wide range of different beams and etalon parameters. As the AAF implementation is known to be experimentally valid [10], this suggests the ABCD etalon model is also experimentally valid.

## 4. Model demonstrations

To demonstrate the practical applicability of the ABCD etalon model, example models of three optical systems using etalons illuminated by Gaussian beams, chosen to be representative of aspects of a range of possible and existing experimental systems, were constructed. These models were used to calculate ITFs and highlight questions of practical significance.

### 4.1 System for illuminating an FP etalon ultrasound sensor

The first model represents a simplified version of a system used to interrogate an FP etalon based ultrasound sensor [27]. In this system, ultrasound waves modulate the thickness of an etalon, resulting in a spectral shift of the ITF. The wavelength of a laser beam monitoring the sensor is tuned to the edge of an ITF fringe, such that the shift in the ITF leads to a change in the reflected beam intensity. In these conditions, ultrasound can be detected via changes in the light intensity reflected by the etalon, and the sensor sensitivity is determined by the first derivative of the ITF at the operating wavelength.

**Fig. 5.**
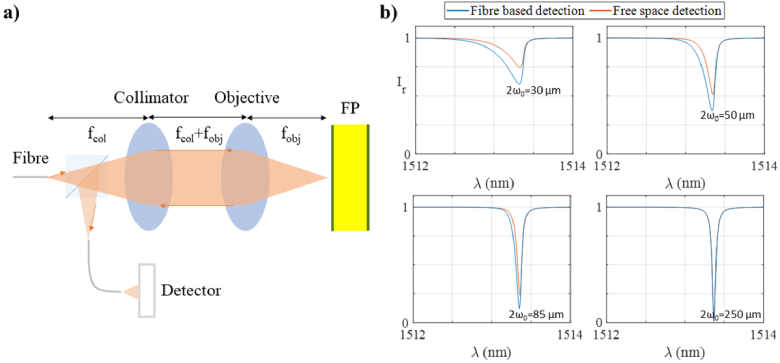
a) Schematic diagram of the modelled optical system consisting of an optical fibre (MFD = 10.4 µm) illuminating an FP etalon (h = 102 µm, n = 1.444, R_1_ = R_2_ = 97%) using a collimator and an objective in a 4f configuration where the reflected beam is directed on to a fibre-coupled detector; and b) Comparison of ITFs in reflection mode for fibre based detection and free space detection (blue and red, respectively), for different spot sizes.

One design feature of this system is that the etalon is illuminated with a focused laser beam in order to provide a relatively small effective ultrasonic detection element, as required for high spatial resolution. To deliver the focussed beam, the system comprises a single mode fibre and two lenses, a collimator of focal length *f_col_* and objective of focal length *f_obj_*, placed in a 4f configuration [21,27] as shown in Fig. 5(a). The beam is thus focussed at a distance *z = (2f_col_ + 2f_obj_)* from the end of the fibre, producing a spot size *2ω_0_ = MFD · f_obj_/f_col_*, where *MFD* is the mode field diameter of the fibre. After interacting with the etalon, the reflected beam is directed on to a fibre-coupled detector as shown in Fig. 5(a).

To model this system, matrices representing the lenses and etalon are multiplied to yield the system matrix:

(15) $$M_{syst,m}=M_{4f}^{\leftarrow }M_{FP,m}M_{4f},$$

where

(16) $$M_{4f}=M_{prop}^{f_{obj}}M_{lens}^{f_{obj}}M_{prop}^{f_{obj}+f_{col}}M_{lens}^{f_{col}}M_{prop}^{f_{col}}=\left[\begin{array}{cc} -f_{obj}/f_{col} & 0\\ 0 & -f_{col}/f_{obj} \end{array}\right].$$

The ITF is then obtained using Eq. (12), with *I_out_* given by Eq. (14) to account for the fibre detection scheme.

This model allows studying the impact of various optical design parameters on the performance of the system. For example, it allows examining the trade-off between the sensitivity and element size simply by changing the ABCD matrices representing the lenses to change the focal spot size of the beam on the etalon. This capability is illustrated in Fig. 5(b), which shows simulated ITFs (blue lines) for different sets of lenses producing four different spot sizes. As expected, the ITFs produced by the smaller spot sizes exhibit lower ITF gradients [10]. Since the sensitivity of the sensor depends on this gradient, the smaller element size is associated with a lower sensitivity. In the cases illustrated in Fig. 5, the ITF maximum gradient is 25, 20, 12 and 7 nm^−1^ when the spot size is 250, 85, 50 and 30 µm, respectively.

Another potential application of this model is comparing free space and fibre detection schemes, both of which have been used in this kind of system [21]. To illustrate this, the model was also used to simulate the ITFs for a free-space infinite detector (red lines) by using Eq. (13) to calculate *I_out_*. Due to the mode filtering of the fibre, the fibre coupled and free space detectors produced different ITFs [25] as shown in Fig. 5(b). For the smaller spot diameters (30, 50 and 85 µm), the fibre-based detection scheme provides a higher visibility and lower FWHM as compared to the free-space detection scheme and therefore yield a higher sensitivity (ITF maximum gradients are 5, 10 and 17 nm^−1^, respectively). However, for the largest spot diameter (250 µm), the ITFs are near-identical indicating that the fibre-based detection scheme confers little discernible benefit in terms of sensitivity (the maximum gradient was 25 nm^−1^ in both cases).

### 4.2 System employing a fibre sensor and a GRIN lens

**Fig. 6.**
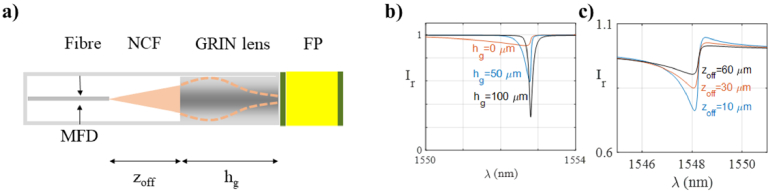
a) Schematic diagram of an FP etalon integrated in an optical fibre illuminated by a diverging beam focussed using a GRIN lens; b) reflection-mode ITFs obtained using the ABCD model for different lengths of GRIN lens (h_g_ = 0 µm, h_g_ = 50 µm and h_g_ = 100 µm); and c) ITFs obtained for different lengths of NCF (z_off_ = 10, 30 and 60 µm). For the simulations, the etalon parameters were n_0_ = 1.444, h_g_ = 102 µm, z_off_ = 10 µm, and MFD = 10.4 µm

The second model is representative of aspects of compact systems featuring fibre-optic etalon sensors. Such systems are used to measure pressures, temperatures, strains and acoustic waves in fields including medical and structural engineering [23,28]. One common configuration is shown in Fig. 6(a). In this configuration, the light is delivered to and from the etalon by an optical fibre. To reduce the beam divergence, the beam is first expanded via its propagation through a section of no-core-fibre (NCF), and then focussed onto the etalon using a graded index (GRIN) lens [29]. For measuring the reflected light, a single-mode fibre coupled detector is assumed. In common with the system described in section 4.1, the spot size affects the ITF, which determines the sensor sensitivity. Here, the spot size is determined by the parameters of the GRIN lens and the length of the NCF, *z_off_*.

The ABCD model is well suited to simulating this system because it can incorporate the GRIN lens (which is not straightforward with other models) and the NCF using existing ABCD matrices defined in Table 1. Specifically, the NCF can be modelled using a matrix *M_prop_*, representing propagation through the homogeneous medium of length *z_off_*. Likewise, the GRIN lens can be modelled by a matrix *M_GRIN_*, determined by the length and gradient of the medium as shown in Table 1. Multiplying these matrices by the FP etalon matrix in the appropriate order yields the system transfer matrix

(17) $$M_{syst,m}=M_{prop}^{z_{off}\leftarrow }M_{GRIN}^{\leftarrow }M_{FP,m}M_{GRIN}M_{prop}^{z_{off}}.$$

Using Eq. (15) to account for the fibre coupled detector, this model can be used to examine the impact of parameters of the fibre-based FP etalon system on the ITF. To illustrate this, two simple studies were conducted. First, to study the role of the GRIN lens, ITFs produced assuming different lengths for the GRIN lens were calculated, plotted in Fig. 6(b). The length of the lens affects the beam divergence and, for a certain length [30], the beam is focussed on the first mirror of the etalon. By minimising the divergence in the cavity, this provides a higher sensitivity compared to illuminating the etalon with a shorter lens or with no lens. In this specific case, as the length of the lens was changed from 0 to 100 µm, the depth and sharpness of the ITF increased [Fig. 6(b)], and its maximum gradient increased from 1 nm^−1^ to 7 nm^−1^.

This model could also be used to simulate the effect of variations of the length of the NCF on the ITF. To illustrate this, ITFs were calculated for three different lengths of NCF and plotted in Fig. 6(c). As expected, different values of *z_off_* produce ITFs with different visibility and finesse. This is because the position of the focal plane after the GRIN lens depends on how much the beam has diverged before reaching the lens.

The above examples illustrate how the ABCD etalon model can be used to study the impact of changing parameters like the lengths of the GRIN lens and NCF on the etalon response. Through similar studies, the model might enable studying the fabrication tolerances on such parameters, and evaluating the impact of misalignments along the optical axis in fibre-based etalon systems.

### 4.3 System with cascaded FP etalons

**Fig. 7.**
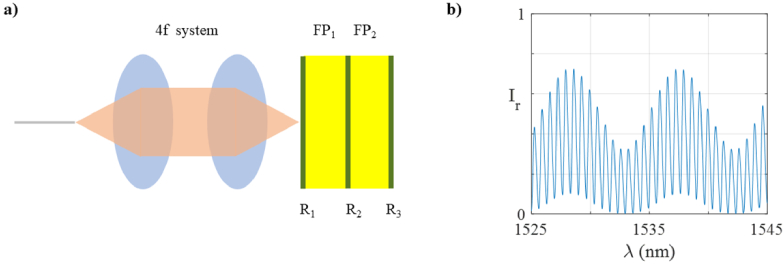
a) Schematic diagram of the modelled optical system consisting of two cascaded FP etalons and a pair of thin lenses in 4f configuration illuminated by a single mode fibre; and b) Simulated ITF in reflection mode of the system, when h_1_ = 100 µm, h_2_ = 400 µm, R_1_ = R_2_ = R_3_ = 20%, n = 1.444, and 2ω0 = 30 µm.

As a third example, a system employing two cascaded etalons is considered [Fig. 7(a)]. Practical systems featuring cascaded etalons (in different specific configurations) exist in the literature and have been used, for example, as sensors for simultaneously monitoring temperature and pressure [31]. Such systems have also been used to generate the optical Vernier effect to increase sensitivity [32]. In the modelled system, the cascaded etalons comprise a series of three mirrors, separated by two parallel cavities made of the same material. A 4f system delivers light from an optical fibre to the etalons and back.

In our ABCD representation of this system, the etalons are represented by two separate transfer matrices, each describing one etalon in terms of its mirror reflectivity, thickness, and refractive index, resulting in *M_FP,m_* [Eq. (9)]. In this case, the beam undergoes *m* round trips inside the first etalon and is then transmitted to the second etalon, where it undergoes *n* round trips. As such, the system matrix can be obtained by multiplying the individual FP etalon matrices together, along with matrices representing the other optics in the system:

(18) $$M_{syst,m,n}=M_{4f}^{\leftarrow }M_{FP2,n}M_{FP1,m}M_{4f},$$

The field distribution of each partial beam, *U_m,n_*, is calculated by means of Eq. (4) using the total transfer matrix. Finally, the partial beams are summed as described in Eq. (11), accounting for the mirror reflectivities of both etalons.

Figure 7(b) shows the predicted ITF of this system, which exhibits rapid oscillations within a more slowly varying envelope. The appearance of these features is expected; while the ITFs of individual etalons are determined by the optical thickness of the cavities, the envelope observed in the response of this system is determined by the free spectral ranges of both etalons as described in Ref. [33]. The model could be used to study the unique features of cascaded etalon systems. For example, when the optical thickness of the etalons are perturbed (e.g., as might occur due to change in pressure in pressure sensing systems), the envelope shift can be greater than the shift of the individual ITFs, increasing the sensitivity [34]. Equally, in other such sensing systems, one of the two etalons can be made to be insensitive to a quantity such as temperature, enhancing the robustness of the device [33]. In each of these conditions, by modelling this system in a modular and intuitive way, the ABCD could provide a suitable tool for investigating the impact of various design parameters such as the thicknesses of the two etalons on the sensitivity and other characteristics.

## 5. Discussion

A model of Gaussian beam propagation through FP etalon systems based on the ABCD transfer matrix method was presented. To validate the etalon model, it was compared to a previously experimentally validated Angular Airy function model [7]. Good agreement was observed, suggesting the ABCD model is accurate. To demonstrate the utility of the model, three optical systems of practical interest were simulated. Simulating these systems required modelling not only the response of an etalon but also the optical components (single mode fibres, focussing lenses, GRIN lenses) used to deliver light to and from it. Modelling such multi-component systems is particularly well served by the ABCD approach due to the modularity of its implementation.

The simplicity of the ABCD method is enabled by certain approximations. These include assuming the system satisfies the paraxial beam approximation, meaning the angular deviation, *θ_d_*, from the optical axis is small enough that *sin(θ_d_)≈θ_d_*. The method also assumes that any lenses are significantly larger than the beam width, and that mirrors can be defined only by their reflection and transmission coefficients, neglecting their thicknesses and, where applicable, their stratified nature. In most systems involving etalons illuminated by Gaussian beams, these approximations are expected to be valid, and therefore not limiting.

In terms of the efficiency of the ABCD etalon model, the time taken to calculate ITFs using the model is determined by the spatial sampling of the beam profile, the number of wavelengths, and the number of round trips. The model was implemented in cylindrical coordinates, which minimises the number of spatial points and speeds up the computational implementation by assuming radial symmetry. For all the ITFs presented in section 3, the simulation time was <1 minute (using a computer with a 64-bit operating system, Intel Core i7-9700 CPU 3.0 GHz processor, 16GB RAM), which is similar to the simulation time using the AAF. Adding extra components (illumination and detection optics) incurs negligible additional computational cost. However, cascading multiple etalons is more time consuming because each etalon matrix has to be iterated.

The model could be extended to broaden its applicability. For example, while it can already account for axial misalignments of the interrogation beam (Section 4.2), modifying the matrices in Table 1 to propagate the beam’s off-axis displacement and slope through the optical system could allow accounting for radial misalignments and rotations of components, as described in Ref. [35]. As well as allowing the model to study tolerances on the misalignments of etalons and their surrounding optics, this could allow investigating the impact of tilted mirrors and thickness nonuniformity in FP etalons [36]. Different illumination beams could also be modelled as, while the current implementation considers Gaussian beams of the lowest order, other beams are also valid within the same framework. For example, by using a different solution to Eq. (3), such as Laguerre-Gaussian beams or Hermite-Gaussian beams, the model could also be adapted to account for higher-order beams [37]. In principle, aberrated beams could also be modelled by decomposing the input wave into a superposition of Gaußlets [38,39] Beyond changing the beams, more complex optical cavities could be modelled. For example, although generic cavity non-uniformities such as those due to mirror roughness cannot be straightforwardly represented, other resonators of certain simple geometries could be. For example, by changing the choice of ABCD matrices used to represent the cavity to use one planar and one spherical mirror, the model could be extended to modelling planoconcave microresonators [40]. As with the presented planar etalon model, such adaptations could be prone to deviation from experiment for a range of reasons, so rigorous validation via experiment or comparison with previously validated models would be required prior to their adoption.

## 6. Conclusion

A model of Gaussian beam propagation through FP etalon systems was presented. The model makes use of the ABCD transfer matrix method, in which the etalon and the optical components to deliver the light to and from the etalon are represented by matrices, and light is propagated through the system iteratively to account for the multiple round trips that light undergoes in the etalon. The model is valid and straightforwardly allows modelling complex multi-component etalon systems. As such, it could aid designing and evaluating a range of etalon systems in fields including astrophysics, biomedical imaging, and civil engineering. It could also be extended in a number of ways, such as to allow simulating off-axis and tilted components, and FP devices of other geometries.

## Data Availability

The code is open-source and freely available in Ref. [41].
